# Laparoscopic left hemihepatectomy guided by real-time indocyanine green fluorescence imaging using the arantius-first approach

**DOI:** 10.1186/s12957-023-03165-9

**Published:** 2023-09-06

**Authors:** Jiaguo Wang, Jie Xu, Kai Lei, Ke You, Zuojin Liu

**Affiliations:** Department of Hepatobiliary Surgery, The Second Affiliated Hospital of Chongqing Medical University, No. 76, Linjiang Road, Yuzhong District, Chongqing, 400010 China

**Keywords:** Laparoscopic left hemihepatectomy, Laparoscopic portal territory hepatectomy, The Arantius-first approach, Real-time ICG fluorescence imaging

## Abstract

**Background and objective:**

Laparoscopic hepatectomy approaches, including major hepatectomy, were rapidly developed in the past decade. However, standard laparoscopic left hemihepatectomy (LLH) is still only performed in high-volume medical centres. In our series, we describe our technical details and surgical outcomes of LLH.

**Methods:**

Thirty-nine patients who underwent LLH in our institute were enrolled in the study. Among these, 13 patients underwent LLH guided by real-time ICG fluorescence imaging using the Arantius-first approach (ICG-LLH group), and the other 26 underwent conventional LLH (conventional LLH group). Demographic characteristics and perioperative data were retrospectively collected and analysed. We compared the technical and postoperative short-term outcomes of the two groups.

**Results:**

There were no significant differences in the demographic or clinicopathological characteristics of the patients in the two groups. ICG-LLH required significantly fewer pringle manoeuvres (1 vs. 3 times, *p* < 0.0001), had a shorter parenchyma dissection time (26 vs. 78 min, *p* < 0.001), and required fewer vessel clips (18 vs. 28, *p* < 0.001). Although there was no significant difference, the ICG-LLH group had less bile leakage (0 vs. 5, *p* = 0.09) and less blood loss (120 vs. 165, *p* = 0.119). There were no significant differences in the overall complication or R0 resection rates between the two groups.

**Conclusion:**

Our data demonstrate that laparoscopic left hemihepatectomy guided by real-time ICG fluorescence imaging using the Arantius-first approach is safe and feasible in selected patients, thus improving the fluency of the surgical procedure and postoperative short-term outcomes.

**Supplementary Information:**

The online version contains supplementary material available at 10.1186/s12957-023-03165-9.

## Introduction

**Fig. 1:**
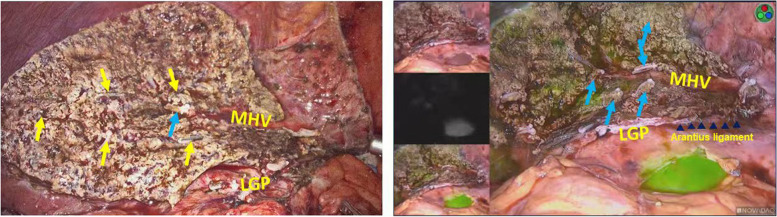
Findings after laparoscopic left hemihepatectomy (LLH). Left figure, the liver resection plane is relatively regular in the conventional LLH; right figure, the liver resection is not a regular plane. Black arrowheads, Arantius ligament; yellow arrows, stump of the Glissonean pedicle; blue arrows, stump of the hepatic vein; MHV, middle hepatic vein; LGP, left Glissonean pedicle

Laparoscopic left hemihepatectomy (LLH) is still the standard operation for treating benign and malignant diseases such as hepatocellular carcinoma, cholangiocarcinoma, and cholelithiasis in the left liver. With nearly 30 years of improvements, the safety and effectiveness of laparoscopic hepatectomy have been verified [1]. In conventional left hemihepatectomy, Rex-Cantlie’s line on the surface of the liver is drawn according to the ischaemic line or anatomical markers, and parenchymal transection is performed along the course of the middle hepatic vein (MHV). However, there is individual variation in the course of the MHV [2]. Small deviations can result in removing too much functional liver tissue or leaving too much nonfunctional liver tissue. The theory of “tumour-bearing portal territory” [3] has confirmed that the actual boundary of the hemispheres of the liver is not a regular plane but an irregular section (Fig. 1). Therefore, conventional left hemihepatectomy is prone to cause injury to the intrahepatic duct system and increase the risk of postoperative complications such as bile leakage, infection, and tumour recurrence. With the advent precise minimally invasive surgery, an increasing number of surgeons are required to not only control surgical trauma as much as possible but also ensure oncologic benefits [4]. Therefore, although LLH is a relatively simple entry-level procedure, standard LLH is still only performed in high-volume medical centres with proficiency in minimally invasive surgery. Given the relatively fixed trocar position and the benefits of laparoscopy in liver surgery, a variety of individualized laparoscopic approaches have been created [5]. Selecting a reasonable surgical approach is essential to ensure a smooth operation process and improve the confidence of the surgeon.

Indocyanine green (ICG) has been widely used in hepatobiliary surgery due to its persistent near-infrared fluorescence (NIRF) properties [6, 7]. At present, the most promising development is that it can be used in anatomic liver resection of the portal territory by staining the target Glissonean pedicle [8]. In this article, we reported the outcomes of Arantius-first LLH under the of real-time guidance of ICG fluorescence imaging. This method has been shown to promote the standardization of surgical procedures, reduce the surgeon’s dependence on laparoscopic surgery experience, and improve surgical fluency.

## Materials and methods

### General information

The clinical data of patients who underwent LLH at the Second Affiliated Hospital of Chongqing Medical University between January 2019 and October 2022 were retrospectively analysed. The inclusion criteria for this procedure were the same as those for conventional LLH. We routinely performed LLH with real-time navigation of ICG staining using the Arantius-first approach after March 2021. All LLH patients were divided into two groups. In the ICG-LLH group, patients underwent LLH with real-time navigation of ICG staining using the Arantius-first approach, and, in the conventional group, patients underwent LLH using the conventional approach (hepatic vein-guided approach (HVGA) with caudo-peripheral direction). Biochemical and radiological examinations (contrast abdominal CT and MRI) were routinely performed in all patients. The demographic and clinicopathological characteristics of the patients (demographic characteristics, perioperative liver function, operation time, number of Pringle manoeuvres, duration of liver parenchyma dissection, intraoperative bleeding, length of postoperative stay, postoperative complications, etc.) were retrospectively reviewed and analysed. Perioperative management was performed in accordance with the enhanced recovery after surgery (ERAS) protocol. The terminology in the article was selected from the expert consensus guidelines published by Gotohda N. et al. [9].

All patients provided informed consent prior to surgery, and this study was approved by the Ethics Review Committee of the Second Affiliated Hospital of Chongqing Medical University.

### Surgical procedure

The patient was placed in a supine position with their legs open and elevated 30°. The operating surgeon stood on the right side, and the assistant stood on the left side. The trocars were inserted according to the 5-hole method. We set an extracorporeal elastic blocking band at the hepatoduodenal ligament to prepare for extracorporeal Pringle’s manoeuvre. A Pringle manoeuvre was performed intermittently during liver parenchyma dissection. The central venous pressure (CVP) remained below 5-cm H2O.

**Fig. 2:**
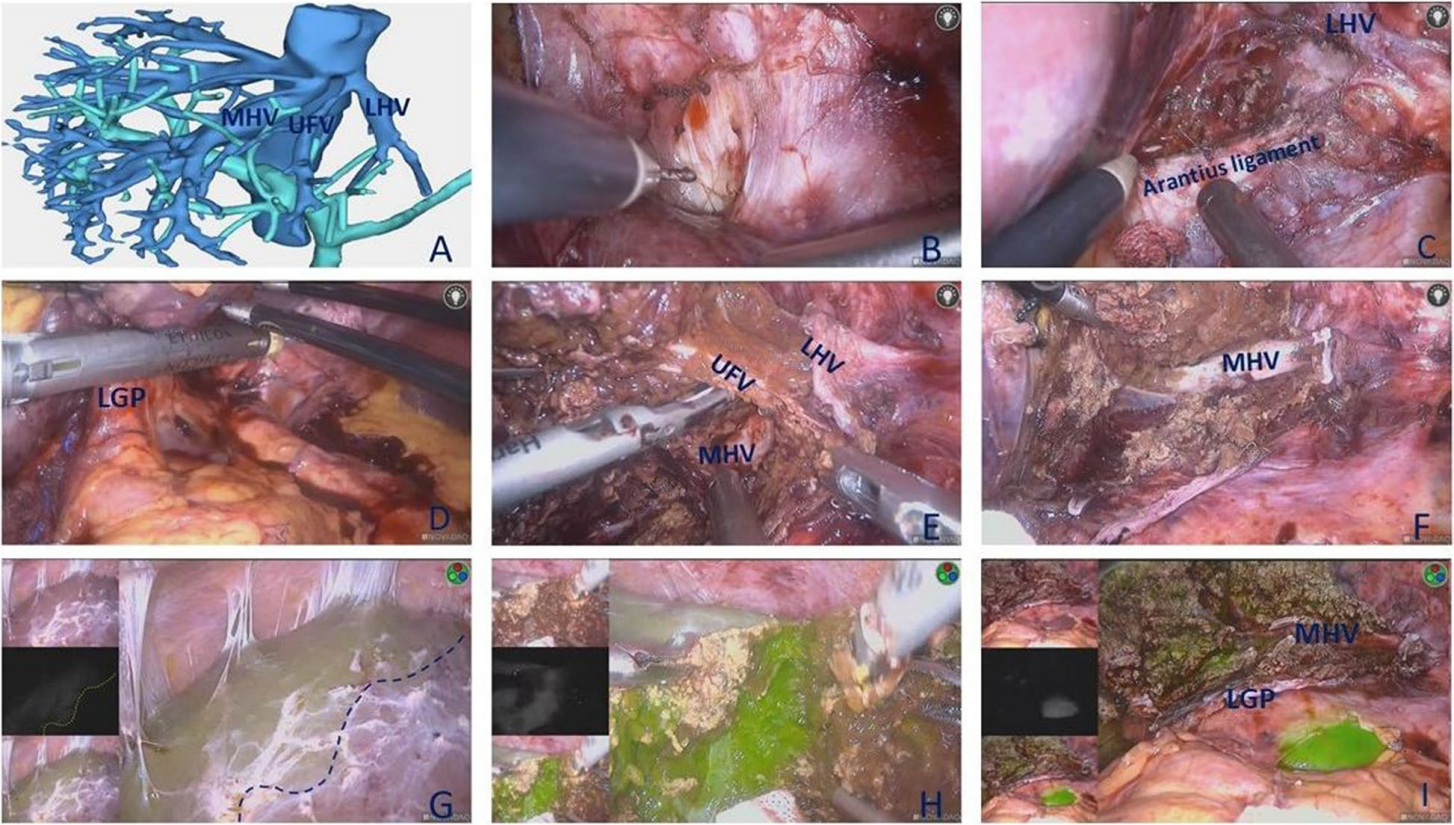
Laparoscopic technique and procedure. **A** preoperative 3D reconstruction. **B** Liver mobilization and dissection of the second hepatic hilum were performed to expose the root of the MHV and left hepatic veins (LHV). **C** The Arantius ligament was used as an anatomic marker in preparing to separate the LHV and the LGP. **D** Exposure and dissection of the LGP. **E** Dividing the LHV and umbilicus fissure vein (UFV). **F** Exposing the MHV towards the cranio-dorsal direction. **G** The definite resection line after injecting 5 mL of ICG (0.025 mg/mL) for negative staining. **H** Parenchymal transection was performed along the fluorescent boundary. **I** Findings after left hemihepatectomy. (Additional file: https://pan.baidu.com/s/1zl0NvdpKm-tNQnshFcfryA?pwd=6kwl)

In the ICG-LLH group, the left hemiliver was mobilized, the round and falciform ligaments were dissected to the second hepatic hilum to expose the root of the MHV, the left hepatic vein (LHV), and the crypt between the MHV and LHV (Fig. 2B); and the left coronary ligament was also divided. After flipping the left lateral lobe, the Arantius ligament was identified between the left lateral lobe and Spiegel’s lobe, the lesser omentum was opened, and the collateral circulation of the left hemiliver was divided. A wide space was opened above the Arantius omentum between the root of the LHV and the left Glissonean pedicle (LGP) without dissecting the liver parenchyma (Fig. 2C). According to the “gate theory” [10], the linear stapler was inserted between gate I and gate III to divide the LGP (Fig. 2D). The dorsal side of the LHV and the umbilical fissure vein (UFV) were opened, whereby the LHV and UFV were easily encircled by communicating this gap to the ventral crypt. The LHV and UFV were divided, and the left hemiliver was completely devascularization (Fig. 2E). The liver parenchyma between the Arantius ligament and the MHV was dissected with a harmonic scalpel (JNJ, Inc., NJ, USA) and a laparoscopic LigaSure (Medtronic, Dublin, Ireland) to expose the MHV in the cranio-dorsal direction, and the hepatic vein branch draining seg. 4 was sequentially ligated and divided (Fig. 2F). Five millilitres of ICG (diluted 1000-fold in 25 mg ICG) was injected through the peripheral veins. The fluorescence-negative regions of the left hemiliver were identified after 5 min in the near-infrared imaging system (PINPOINT, Stryker, Canada) (Fig. 2G). The liver parenchyma was rapidly dissected with both a harmonic scalpel and a laparoscopic LigaSure along the demarcation of the ICG fluorescence, and there were no obvious Glissonean pedicles on the demarcation of the ICG fluorescence (Fig. 2H). The presence or absence of bleeding and bile leakage were carefully checked in the near-infrared imaging system (Fig. 2I).

The patients in the conventional LLH group were prepared in a similar manner as the patients in the ICG-LLH group. The resection line of the liver surface was planned according to the ischaemic line or anatomical markers, and the parenchyma was transected along the course of the MHV. Parenchymal dissection was performed using the “small mouth clamp” with both a harmonic scalpel and a laparoscopic LigaSure. Small vessels and bile ducts less than 3 mm were directly disconnected by a harmonic scalpel and laparoscopic bipolar electrocoagulation, and those greater than 3 mm were clamped by hem-o-lok or titanium clips.

### Statistical analysis

Statistical analyses were performed using SPSS version 26.0 (IBM SPSS, Inc., Chicago, IL, USA). Continuous variables with a normal distribution were described with the mean value (standard deviation (SD)). Continuous variables without a normal distribution were described with the median (interquartile range (IQR)). Counts and percentages were used to summarize categorical variables. Differences between the groups were compared by using either the independent samples *t*-test or the Mann–Whitney *U*-test, and categorical variables were compared with the chi-square test.

## Results

From January 2019 to October 2022, a total of 39 patients underwent LLH in our hospital. Thirteen of these patients underwent LLH under the real-time guidance of indocyanine green fluorescence imaging using the Arantius-first approach. The demographic and clinicopathological characteristics of the patients are summarized in Table 1. There were no significant differences in age, history of abdominal surgery, basic disease, preoperative Child–Pugh classification, aetiology, or tumour load between the two groups. Indications for anatomical liver resection were hepatocellular carcinoma (HCC), intrahepatic cholangiocarcinoma (ICC), colorectal cancer liver metastasis, and cholelithiasis.

**Table 1:** Demographic and clinicopathological characteristics of the patients

Variables	Conventional LLH (*n* = 26)	ICG-LLH (*n* = 13)	*p*-value
**Age, years**	57.8 (10.4)	57.2 (11.3)	0.883
**HAS**	3 (11.5%)	2 (15.4%)	0.735
**DM**	5 (19.2%)	4 (30.8%)	0.420
**Hypertension**	4 (15.4%)	3 (23.1%)	0.555
**HD**	7 (26.9%)	1 (7.7%)	0.161
**BLD**	22 (84.6%)	10 (76.9%)	0.555
**Aetiology**			
**HBV**	19 (73.1%)	9 (69.2%)	0.778
**HCV**	1 (3.8%)	1 (7.7%)	
**Alcohol**	2 (7.7%)	1 (7.7%)	
**Cholelithiasis**	2 (7.7%)	1 (7.7%)	
**CRLM**	2 (7.7%)	2 (15.4%)	
**Child–Pugh classification**			
**A**	23 (88.5%)	12 (92.3%)	0.709
**B**	3 (11.5%)	1 (7.7%)	
**ALT (IU/L)**	26 (20–45)	43 (27–62)	0.136
**AST (IU/L)**	41 (30–62)	38 (24–53)	0.348
**Pathology**			
**HCC**	19 (73.1%)	10 (76.9%)	0.986
**ICC**	3 (11.5%)	1 (7.7%)	
**Cholelithiasis**	2 (7.7%)	1 (7.7%)	
**CRLM**	2 (7.7%)	1 (7.7%)	
**Tumour number**			
**Single**	20 (76.9%)	10 (76.9%)	0.453
**Double**	3 (11.5%)	1 (7.7%)	
**Triple**	0 (0%)	1 (7.7%)	
**Multiple**	1 (3.8%)	0 (0%)	
**Tumour size**	45 (31–65)	51 (45–62)	0.365
**Vascular invasion**	6 (23.1%)	3 (23.1%)	1.000

*HAS* history of abdominal surgery, *DM* diabetes mellitus, *HD* history of drinking, *BLD* background of liver disease, *HBV* hepatitis B virus, *HCV* hepatitis C virus, *CRLM* colorectal cancer liver metastasis, *HCC* hepatocellular carcinoma, *ICC* intrahepatic cholangiocellular carcinoma

The comparison of the intraoperative data and postoperative short-term outcomes between the two groups is shown in Table 2. The ICG-LLH group had better intraoperative data than the conventional LLH group. ICG-LLH was associated with significantly fewer Pringle manoeuvres than conventional surgery (1 vs. 3 times, *p* < 0.0001). ICG-LLH had a shorter parenchyma dissection time (78 vs. 26 min, *p* < 0.001) and required fewer clips (hem-o-lock clip, absorbable ligating clip, and titanium clips) (18 vs. 28, *p* < 0.001) than the conventional LLH group. Although there was no significant difference, the ICG-LLH group had a shorter operation time (180 vs. 195 min, *p* = 0.425) and less blood loss (120 vs. 165, *p* = 0.119) than the conventional LLH group. In terms of postoperative outcomes, postoperative ALT and AST levels, overall complication and R0 resection rates, and length of postoperative hospital stay were comparable between the two groups. There was no intraoperative conversion to laparotomy, deaths within 30 postoperative days, or postoperative haemorrhage in either group. In the conventional LLH group, five patients suffered postoperative bile leakage, three of whom were in better conditions after long-term drainage, and the conditions of the remaining two improved after their biliary pressures were reduced through ERCP. In the ICG-LLH group, we accidentally divided the MHV in one patient, but the patient had no serious postoperative complications.

**Table 2:** Intraoperative data and postoperative outcomes

Variables	Conventional LLH (*n* = 26)	ICG-LLH (*n* = 13)	*p*-value
**OT**	195 (170–248)	180 (173–215)	0.429
**PM**	3 (2–3)	1 (1–2)	** < 0.001**
**PDT**	78 (69–86)	26 (23–33)	** < 0.001**
**Clips**	28 (2–35)	18 (17–23)	** < 0.001**
**Blood loss**	165 (128–200)	120 (90–200)	0.119
**Conversion**	0	0	1.000
**Bile leakage**	5 (19.2%)	0 (0%)	0.090
**Haemorrhage**	0	0	1.000
**ALT (IU/L)**	43 (25–60)	56 (27–99)	0.270
**AST (IU/L)**	70 (37–140)	129 (42–178)	0.456
**Clavien–Dindo grades 1–2**	21 (80.8%)	11 (84.6%)	0.768
**Clavien–Dindo grades 3–4**	4 (15.4%)	0 (0%)	0.135
**30-day mortality**	0	0	1.000
**R0**	26 (100%)	13 (100%)	1.000
**PHS**	7 (6–8)	7 (7–9)	0.913

*OT* operation time, *PM* pringle manoeuvre, *PDT* parenchyma dissection time, *PHS* postoperative hospital stay

## Discussion

The safety and feasibility of LLH have been reported previously, but laparoscopic major hepatectomy remains technically challenging and still requires a certain number of cases to master the learning curve [11–13]. Anatomical liver resection was proposed by Makuuchi et al. in 1985 and refers to the systematic removal of primary lesions and the corresponding tumour-bearing portal venous territories [3]. Because of the unique oncological characteristics of HCC, anatomical liver resection could theoretically improve the tumour prognosis. Moreover, the preservation of residual liver parenchymal vessels and bile ducts can reduce the risk of postoperative complications, which has been confirmed by previous reports [14]. Given that the hemiliver boundary is patient specific, conventional LLH, anatomical portal territory hepatectomy, and full devascularization of specimens may not be a good first-line treatment for ischemia in all cases, especially in patients with cholelithiasis with liver atrophy or adhesion of the liver capsule. Second, ischaemic demarcation is merely the resection line of the liver surface, and there is no ischaemic marker within the liver parenchyma; however, ICG-guided liver resection can effectively overcome this difficulty. ICG fluorescent navigation provides technical support for achieving anatomical liver resection. Negative staining was considered a favourable technique for hemihepatectomy using Takasaki’s extrahepatic Gleason approach [15]. Because of technical limitations, conventional anatomical liver resection can only identify the landmark hepatic vein to achieve an approximate portal territory hepatectomy, and accurate creation of the hemiliver boundary is the key to the success of the operation. The liver surface resection line is the Rex-Cantlie line drawn according to the hemihepatic ischaemic line or anatomical landmarks, and the resection line within the liver parenchyma is mainly dependent on the course of the MHV. However, the actual boundary of the hemiliver is not a regular straight line but an irregular curve (Fig. 1). This was also confirmed in our study. ICG-LLH required fewer clips (hem-o-lok clip, absorbable ligating clip, and titanium clips) (18 vs. 28, *p* < 0.001) than conventional LLH, which also confirmed that fewer vascular and bile duct systems were encountered during liver parenchyma dissection in ICG-LLH (Fig. 1). Theoretically, there are no Glissonean pedicles in an intersegmental plane, and our study also confirms that ICG-labelled liver transections are more consistent with physiological liver fissures (Fig. 1).

Furthermore, biliary leakage after liver resection is a common complication, with an incidence ranging from 4.0 to 9.8% in the literature [16]. The effectiveness of using the ICG fluorescence navigation system for intraoperative evaluation of small bile duct leakage in the liver section has been demonstrated [15]. In our study, although there was no significant difference, the incidence of biliary leakage was higher in the conventional LLH group (19.2% vs. 0, *p* = 0.090). Otsuka, Y. et al. [17] found that the ICG fluorescent navigation system can help detect bile leakage in the early intraoperative period to provide timely repair. In our study, postoperative biliary leakage mainly occurred in patients with cholelithiasis, which showed the wider application prospects of fluorescent navigation systems in treating biliary diseases. Moreover, some literature has confirmed that the Pringle manoeuvre can improve the tolerance of the remnant liver to ischaemia and promote liver regeneration [18, 19]. The Pringle manoeuvre is a favourable technique to prevent and control bleeding during LAR; however, some studies have also shown that the Pringle manoeuvre might have a negative impact on oncological outcomes [20–22]. Prolonged blockade may exacerbate an ischaemic reperfusion injury. In our study, ICG-LLH was associated with significantly fewer Pringle manoeuvres (1 vs. 3 times, *p* < 0.0001) and a shorter parenchyma dissection time (78 vs. 26 min, *p* < 0.001) than the conventional group. All of these findings confirm the safety and feasibility of our approach to LLH.

**Fig. 3:**
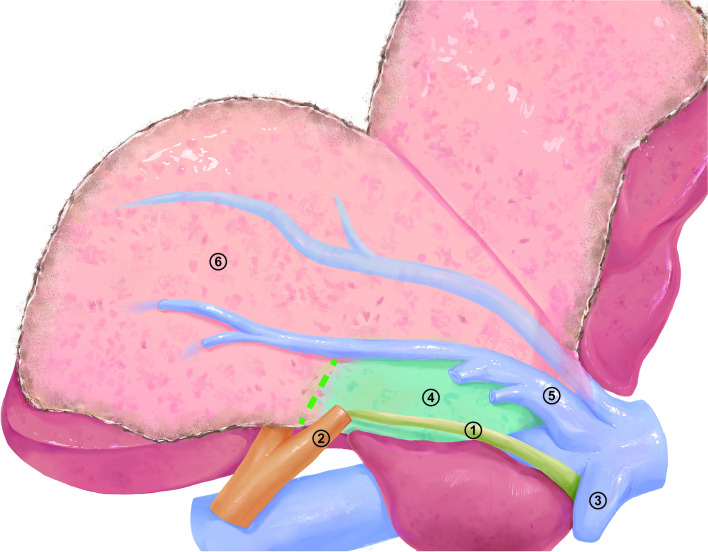
Diagram of the surgical procedure. Step 1, creation of a wide space above the Arantius ligament between the root of the LHV and the LGP without dissecting the liver parenchyma; step 2, exposure and dissection of the LGP; step 3, dissection of the LHV from the UFV; step 4, dissection of the liver parenchyma between the Arantius ligament and the MHV; step 5, exposure of the MHV; step 6, transection of the parenchyma along the fluorescent boundary

Another thing worth mentioning is the ability to control hepatic inflow and outflow before liver parenchyma dissection. Various approaches to LLH have been reported [23]. Moreover, laparoscopic anatomical portal territory hepatectomy with real-time navigation of ICG-negative staining requires control of the target pedicle by an extrahepatic approach without parenchymal destruction or an intrahepatic approach with minor liver transection before liver parenchyma dissection. After a comprehensive evaluation, the Arantius-first approach [24] was determined to be the preferred technique for LLH (Fig. 3). The advantages of this approach are summarized as follows:

1. The left hemiliver can be fully mobilized, and all the collateral circulation of the left hemiliver can be divided to prepare for negative staining in the later stage.
2. In accordance with the “en bloc” principle, the LGP, LHV, and UFV were preferentially divided before liver parenchyma dissection, and the specimen was thoroughly devascularized.
3. The liver parenchyma above the Arantius ligament was opened between the root of the LHV and the LGP. The posterior aspect of the LGP was exposed, and sufficient space behind the LGP was reserved. The LGP was safely divided on the ventral side of the Arantius ligament.
4. The MHV was preferentially exposed to avoid “tenting” the hepatic vein [25] before liver parenchyma dissection.
5. After devascularization of the specimen, the liver parenchyma was quickly dissected along the fluorescence boundary, thereby reducing the number and frequency of pringle manoeuvres and reducing the risk of ischaemia–reperfusion injury.

Of course, this approach also has limitations; it is difficult to upturn the left lobe of the liver when there is obvious left liver hyperplasia, and the tumour volume is large. Moreover, it is sometimes difficult to distinguish the relationship between the MHV and the UFV. In our case series, we also accidentally divided the MHV of a patient. By evaluating several cases, we found that the MHV still had a caudo-dorsal course, while the UFV had a caudo-ventral course when the left lateral lobe was flipped, which is relatively easy to distinguish between the two. The timing and dose of ICG administration for obtaining a higher specificity of ICG fluorescence imaging are also worth mentioning. A systematic review [26] showed that ICG fluorescence visualization is different among fluorescent imaging systems due to tremendous bias in the standardization of the dose and timing of ICG administration. Our experience showed that the ICG dose (5 mL, 0.025 mg/L) should be as small as possible to achieve functional staining, which can greatly reduce fluorescence contamination.

There are also limitations in this study. This was a retrospective case–control, small-sample, single-centre study with a short-term follow-up. Therefore, it is necessary to conduct multicentre, large-sample randomized controlled studies with long-term follow-ups to confirm our results.

## Conclusions

The Arantius-first approach can effectively control left hepatic inflow and outflow before liver parenchyma dissection and can improve the success rate and staining effect of ICG fluorescent negative staining. This procedure has been previously attempted, and its process is the closest to the theory of anatomical portal territory hepatectomy.

### Supplementary Information

**Additional file 1: Video.** Laparoscopic Left Hemihepatectomy.

## Data Availability

The original contributions presented in the study are included in the article or supplementary material, and further inquiries can be directed to the corresponding author.
